# Evaluation of Surface Treatment for Enhancing Adhesion at the Metal–Composite Interface in Fibre Metal-Laminates

**DOI:** 10.3390/ma15176118

**Published:** 2022-09-03

**Authors:** Magda Droździel-Jurkiewicz, Jarosław Bieniaś

**Affiliations:** Department of Materials Engineering, Faculty of Mechanical Engineering, Lublin University of Technology, Nadbystrzycka 36, 20-618 Lublin, Poland

**Keywords:** fibre-metal laminates, surface treatment, aluminium, titanium, interface, adhesion

## Abstract

The paper presents the issues of metal surface treatment in fibre metal laminates (FML) to obtain high adhesion at the metal–composite interface. Aluminium 2024-T3 and titanium Grade 2 were analysed. The metal surface modifications were carried out by mechanical (sandblasting, Scotch-Brite abrasion), chemical (P2 etching, phosphate-fluoride process), electrochemical (chromic and sulphuric acid anodizing), and plasma treatment, as well as the application of sol-gel coatings. In terms of surface geometry, the analysis included roughness and 3D surface topography examination. The morphology was examined using scanning electron and atomic force microscopy. The surface free energy and its components (polar and dispersive) were determined using the Owens–Wendt method. The novelty of this study is the determination of the effect of different surface treatments on the surface free energy, topography, and morphology in terms of the possible appropriate adhesion in fibre metal laminates. Chromic acid anodizing is still the most effective in enhancing the expected adhesion. A suitable technique may be the use of P2 etching of aluminium. It results in low roughness, numerous micro-irregularities, and the presence of porosity. The obtained test results show that the application of sol-gel coating increases the surface free energy and may increase the adhesion.

## 1. Introduction

Currently, there are an interesting group of materials, known as fibre metal laminates (FML), which consist of alternating metal and composite layers. They are characterized by low density, high static, fatigue strength [1], impact [2,3], and corrosion resistance [4]. 

In FMLs, the most commonly used materials are aluminium alloys (GLARE^®^: glass-based laminates, or CARALL^®^: carbon-based laminates) and titanium for hybrid titanium composite laminates (HTCLs). The main application of fibre metal laminates includes aerospace applications. They are widely used for structural materials for aircraft parts, such as fuselage frames, certain doors, wings, aircraft tails, and so on [5]. For example, approximately 380 m^2^ of GLARE laminates have been used in the Airbus A380.

One of the most important issues in FMLs remains the metal surface treatment to receive an appropriate adhesion at the metal–composite interface. It determines the obtaining of a combination of high strength and quality joint, with increased resistance to interlayer cracking and environmental conditions [6,7]. Moreover, it performs a crucial role in stress transfer between metal and composite layers [4,8,9,10].

Nowadays, various methods of aluminium and titanium surface preparation, such as mechanical, chemical, and electrochemical, are used in FML laminates. Each of the methods, more or less, change the metal structure and its morphology, which in turn affects the adhesion at the metal–composite interface. Mechanical methods, including grit blasting and mechanical abrasion, change the surface topography and increase the surface roughness, as well as the interaction between the metal and the composite [11]. These methods allow one to obtain a stronger initial adhesion, which is associated with an increase in the surface energy and mechanical interlocking between metal and composite [12,13,14], due to the introduction of the peak-and-valley surface structure [15]. Authors [14] investigated the influence of aluminium surface roughness on the metal–composite interfacial strength in FMLs. They summarized that increasing the surface roughness increases the bonding strength. They also revealed that the surface with the highest roughness provides high adhesion strength at the Al/CFRP interface. Yao et al. [16] have analysed the influence of two different mechanical methods, abrasion, and grit blasting on the metal composite interface in FMLs. Their results showed that grit blasting can effectively enhance the interfacial fracture toughness of STL/CFRP laminates. In another study [17] authors revealed that sandblasting is worse than micro-arc oxidation (MAO) and laser ablation in the case of the adhesion at the Al/CFRP interface.

The use of chemical treatment leads to the surface cleaning of brittle oxides and the formation of a homogeneous oxide layer with some micro-roughness [15]. It is possible to develop the surface with high physicochemical activity, concerning the applied bonding substance [18]. Etching can provide good initial strength and durability of the adhesive bonding, intermediate between mechanical and electrochemical methods [19,20]. Authors [18] tested the influence of modified FPL-etch and P2-etch on the mechanical behaviour of CARALL laminates. They concluded that P2 etching resulted in poor interfacial bond strength between the metal and the carbon composite. Prolongo and Urena [21] noted that P2 etching modifies the surface properties of the 2024 aluminium alloy, by obtaining a porous structure. Due to this, it was possible to achieve the highest adhesion for epoxy-aluminium in the FMLs, concerning FPL-etching, abrasion, or alkaline treatment. Aghamohammadi et al. [22] indicated that alkaline etching produces a rough and flake-like surface; nevertheless, it does not improve adhesive bonding. Furthermore, FPL-etching produces a surface structure similar to sulphuric acid anodizing. Gonzalez-Canche et al. [10] tested the use of three different treatments (sanding, degreasing, and NaOH treatment), and their results showed that the best interfacial adhesion in thermoplastic-based fibre metal laminates was achieved after NaOH treatment, due to obtaining cleaner, rougher, and more wettable surfaces. 

For FMLs electrochemical treatments are widely used in metal surface treatment. These methods allow for the formation of a porous, thin oxide layer with an increased surface area (with a high degree of micro-roughness), as well as an increased number of polar groups on the metal surface. Thanks to this, it is possible to infiltrate the polymer and increase the mechanical and chemical adhesion at the metal/composite interface [11,22,23,24]. Literature data show that the best-known and widely used method of surface treatment is chromic acid anodizing [5,7,25,26,27,28]. However, it has an adverse influence on the environment and humans because of hexavalent chromium presence. Authors [25] investigated the surface morphology depending on the different anodizing processes. They summarized that after chromic or phosphoric acid anodizing and FPL-etching, the surface morphology is similar. They are diverse in terms of pore diameter. Simultaneously, after FPL-etching and phosphoric acid anodizing, the layer density and the surface roughness was lower than in the case of chromic acid anodizing. Literature data show that in the surface treatment for fibre metal laminates, chromic acid anodizing can be replaced by phosphoric acid anodizing. Due to the possibility of receiving a porous oxide layer with nanometre scale and pores uniform pits, which guarantee good adhesion at the metal–composite interface [27,28]. Research on titanium conducted by He et al. [26] shows that the connection of the anodizing process with sandblasting, etching, and or annealing allows the obtaining of a very developed surface, produced from nano- to macro-scale hierarchical structure. 

Currently, it is desirable to conduct research on innovative methods for surface treatment, including plasma treatment [29,30] or sol-gel methods [31,32,33]. The plasma is a promising environmentally friendly method for metal surface preparation concerning increasing the bonding in FMLs. Works [30,34,35] indicate that it allows the removal of contamination and activates the metal surface chemical bonds to improve adhesion [34]. At the same time, it was noted that this method does not change the surface morphology [30]. Authors [36] observed that the use of atmospheric plasma causes the removal of weak boundary layers and surface hydrocarbons. The aluminium surface became more hydrophilic and has higher surface-free energy. This provides excellent adhesion at the metal–polymer interface. The author’s stated that it was achieved due to the surface cleaning and activation without mechanical anchorage. Williams et al. [34] noted that the use of atmospheric pressure helium/oxygen plasma is an effective technique for surface activation prior to the bonding of the aluminium. Moreover, they observed that an even better adhesion can be achieved by combining atmospheric plasma with sol-gel coating and primers. Similar observations were noted for SS/CFRP laminates, where authors [35] after lap shear tests observed an increase in bond strength after plasma activation. Low-temperature, atmospheric-pressure plasma can be an alternative method to chemical etching or mechanical abrasion [35]; whereas, Lin et al. [37] noted that oxygen plasma treatment is not as efficient as nitrogen plasma in adhesive bonding. They stated that nitrogen plasma as a surface treatment in Al/Gf/PP laminates results in higher properties, better than that after phosphoric anodic oxidation. It was explained by combining chemical interlocking with a functional group mechanical interlocking due to a rougher surface.

Sol-gel coatings may provide good adhesion at the metal–composite interface by forming a chemical connection. It was proved that they may assure good bonding at the metal–composite interface in FMLs because epoxy-silane GPS forms reactive organic groups for bonding with an adhesive, and TPOZ (zirconium n-propaxine) reacts with aluminium substrate to produce a covalent chemical bond [38,39]. Liu et al. [40] observed that thin sol-gel coatings resulted in good adhesion due to full cross-linking in comparison to the thicker ones. Cobb et al. investigation [41] showed that sol-gel in conjunction with an adhesive resulted in very good fatigue crack growth resistance and fracture toughness for FMLs based on titanium. Similar observations were noted by Ardila-Rodríguez et al. [33]. Authors showed that the use of a sol-gel coating, but also corrosion inhibition primer resulted in an improvement in the initial adhesion. One potential application of the sol-gel coating on aluminium alloys is to replace the conventionally widely used chromate conversion or primer coating [42]. Authors [43] investigated the structural epoxy bonding using grit blasting in combination with AC-130 sol-gel of aluminium, stainless steel, and titanium. They observed that this method provided durable bonding for aluminium alloy 5053 without the need for using a primer. The use of the sol-gel method may result in good bonding at the metal–composite interface in FMLs due to a covalent chemical bonding. However, when using sol-gel layers for titanium and stainless steel, it is necessary to apply an additional layer of primer to obtain high adhesion.

Based on the state of the art it can be stated that the metal surface treatment plays a crucial role in shaping the metal–composite interface, and thus the properties of FMLs. Therefore, it is necessary to perform research in the field of modification and characterization of the metal surface in the aspect of obtaining appropriate adhesion at the interface between metal and composite layers and increasing the state of knowledge in this area. The paper presents the characteristics of the aluminium and titanium surface subjected to mechanical, chemical, and electrochemical methods, as well as plasma treatment and sol-gel coating. The relationship between the metal surface structure and possible adhesion at the metal–composite interface is presented. The topography and morphology characteristics of the surface, depending on the surface treatment and their physicochemical properties, such as surface free energy, are presented. 

## 2. Materials and Methods

### 2.1. Materials and Surface Treatments

**Figure 1:**
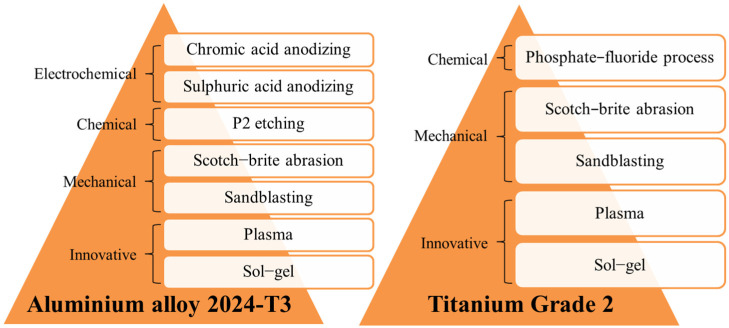
The scheme of aluminium and titanium surface treatments.

Two types of materials, aluminium alloy 2024-T3 and titanium Grade 2, were used to compare different surface treatments. Aluminium and titanium were prepared using mechanical, chemical, electrochemical, and other innovative types of surface treatments, including sol-gel and plasma. Figure 1 presents all surface treatments used during the investigation.

A detailed description of the carried out surface preparation methods is presented in Table 1.

The process of producing FML laminates based on pre-impregnates is carried out in accordance with the autoclave method. The parameters of the subsequent stages of the curing process (temperature, time, pressure, vacuum) depend on the type of composite and the thickness of the laminate. Based on the author’s and co-workers’ research [6], it was noted that due to full control of the autoclave process important parameters, particularly pressure, it is possible to obtain a low-void laminate. It enables the formation of laminates without the porosities and without delamination at metal–composite interface and in the polymer layer.

### 2.2. Surface Characterization

Aluminium and titanium were investigated based on the geometric and morphology analysis, determination of the surface free energy (SFE), and its components. For comparison purposes, selected surfaces were also analysed with a primer layer applied.

The analysis of the surface stereometric parameters was made using 2D/3D methods, profilographometer T8000 RC120-400 (Hommel-Etamic, Villingen-Schwenningen, Germany). To characterize the aluminium and titanium depending on various treatment methods, the data of surface roughness values were obtained by four measurements, two parallel to the rolling direction and two perpendicular to the rolling direction. The calculations were made using Statistica software. The measuring distance was 2 mm. The following parameters were determined as *R_a_*—average roughness, *R_p_*—maximum profile peak height, *R_v_*—maximum profile valley depth, *R_z_*—average maximum height of the profile, *R_t_*—maximum height of the roughness profile, *R_q_*—square root of the roughness. Moreover, surface topography in nano- and micro-scale was scanned in non-contact mode using an atomic force microscope, MultiMode 8 (Bruker, Billerica, MA, USA).

The Owens–Wendt method was used to determine the surface-free energy. The SFE value was calculated based on the measurement of the contact angle of the tested surfaces by measuring liquids with known values of the surface free energy $\left(\gamma_{s}\right),$ and their polar $\left(\gamma_{s}^{p}\right)$ and dispersion components $(\gamma_{s}^{d}$). Distilled water and dijodomethane were used as measuring liquids. Drops of measurement liquids with a fixed volume of approximately 4 μL were automatically applied. Surface-free energy was the sum of the dispersion and polar components. The wettability tests were carried out using a PGX contact angle analyser with the computer image analysis software (Surfaceware software).

Surface characterization was performed using scanning electron microscopy, (NovaNanoSem 450, FEI, Lincoln, NE, USA). In the analysis of the structure of the oxide layers, the method of sample preparation based on the ion etching process (cross-sections) and the observation of fractures after impact tests in the environment of liquid nitrogen were used. Depending on the requirements, the samples were also vaporized with a layer of gold or platinum.

## 3. Results and Discussion

### 3.1. Roughness and Topography

**Figure 2:**
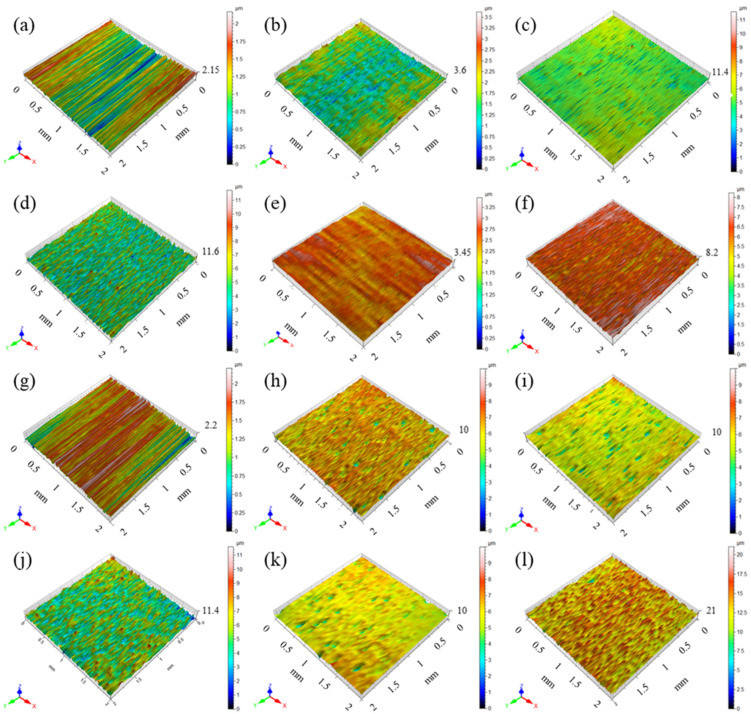
The 3D roughness profiles for aluminium alloy: delivery state (**a**), Scotch-Brite abrasion (**b**), chromic acid anodizing (**c**), sandblasting (**d**), P2 etching (**e**), sulphuric acid anodizing (**f**), plasma (**g**), and titanium: delivery state (**h**), Scotch-Brite abrasion (**i**), phosphate-fluoride process (**j**), plasma (**k**), sandblasting (**l**).

**Figure 3:**
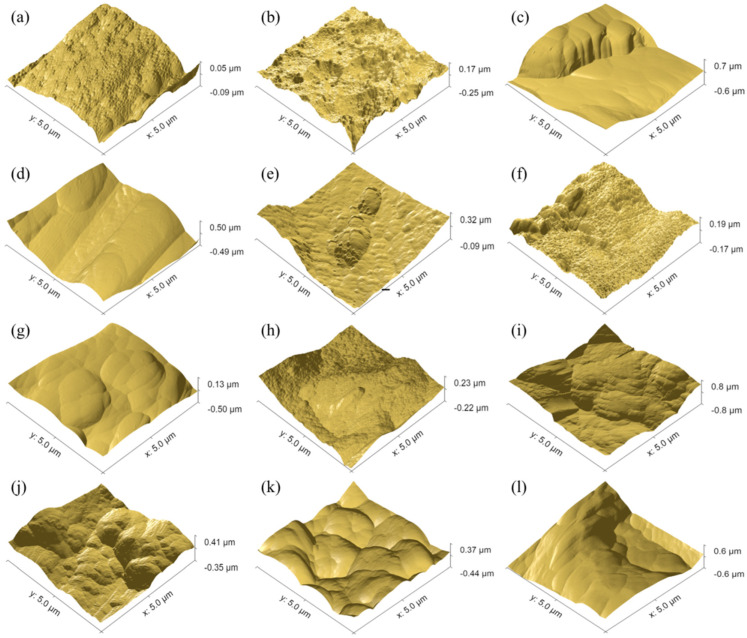
Surface topography of aluminium degreased only (**a**), P2 etching (**b**), sandblasting (**c**), Scotch-Brite abrasion (**d**), plasma (**e**), chromic acid anodizing (**f**), sulphuric acid anodizing (**g**), and titanium: degreased only (**h**), sandblasting (**i**), Scotch-Brite abrasion (**j**), phosphate-fluoride process (**k**), plasma (**l**).

The determined roughness parameters for aluminium and titanium depending on various surface treatment methods are presented in Table 2 and Table 3. However, Figure 2 presents 3D roughness profiles for aluminium and titanium, and Figure 3 shows characteristic AFM roughness profiles.

The analysis of the results shows the dependence of the roughness parameters on the surface treatment method of aluminium and titanium. It was noted that the highest average roughness (*R_a_*), as the primary roughness parameter, was for aluminium after sandblasting (*R_a_* = 0.78 µm). On the other hand, the lowest value was for P2 etching (*R_a_* = 0.07 µm). It seems that the surface may have some micro-roughness, which confirms 3D profiles and AFM results. Where some characteristic cavities on the surface were observed (see Figure 2e and Figure 3b). An equally low roughness value was for aluminium subjected to chromic acid anodizing (CAA), (*R_a_* = 0.17 µm), while the intermediate values were after sulphuric acid anodizing (SAA), (*R_a_* = 0.39 µm). Simultaneously, it was observed that chromic acid anodizing causes the most homogenous surface (see Figure 2c and Figure 3f). It is characterized by micro irregularities with a high degree of uniformity of their distribution on the surface. Whereas, in comparison to CAA, after SAA, the surface had higher roughness, with the presence of areas with significant geometrical diversity (see Figure 2c,f).

It should be noted that for plasma treatment and Scotch-Brite abrasion, the roughness *R_a_* value was at a low level (see Table 2 and Table 3). For aluminium, they were approx. 0.19 µm, and for titanium from 0.28 µm to 0.38 µm. It was noted that in both cases these values are close to the delivery state of the tested metals (see Table 2 and Table 3). Simultaneously, after plasma treatment no significant differences in the surface profilometry were observed, and the metal surface was quite smooth (see Figure 2 and Figure 3). They have a cleansing nature, as it was shown by [34,35]. Only Scotch-Brite abrasion has a slight influence on the change of the geometrical characteristics of the surface (see Figure 2 and Figure 3). The analysis of the other roughness parameters of the aluminium surface showed that they have a similar tendency as in the case of average roughness. In the case of *R_q_*, *R_t_*, *R_z_*, and *R_p_* it was also noted that the highest parameters were for sandblasting. The lowest for P2 etching, equally low for CAA, and intermediate for SAA (see Table 2). 

In the case of titanium, the highest roughness was for sandblasting (*R_a_* = 1.14 µm). Similarly, it was after the phosphate-fluoride process (*R_a_* = 0.98 µm). High roughness for aluminium and titanium after sandblasting is confirmed by the numerous rises and grooves of the surface (see Figure 2d,l and Figure 3c,i). In the case of titanium after phosphate-fluoride (see Figure 2j and Figure 3k), the surface is irregular, characterized by high variability, many grooves, and elevations with significant geometrical differences. As with the analysis of other aluminium parameters. For titanium, it was observed that in the case of *R_q_*, *R_t_*, and *R_p_*, as well as *R_v_* parameters, the tendency was the same as it was in average roughness. The highest values were for sandblasting and phosphate-fluoride process treatment.

The research shows that depending on the surface treatment methods, different types of surface structures were obtained. According to the literature data, there are distinguished three types of titanium surface roughness [44]. The first group indicates that the surface has little micro- or macro-roughness, while the second group has more macro-roughness and a small amount of micro-roughness. The third group is characterized by no macro-roughness but a high amount of micro-roughness, and it is considered to produce a surface with the best durability. Taking into consideration the investigated surface treatments, it seems that group I includes a phosphate-fluoride process and group II titanium after sandblasting. In our research, we can also apply this type of classification to the aluminium surface treatment methods. According to the authors, the I group includes sulphuric acid anodizing, the II group sandblasting, and the III group, due to its structure, includes chromic acid anodizing and P2 etching. As previously emphasized, plasma and Scotch-Brite have cleansing properties. These characteristic types of surface treatments and the obtained surface topography have a significant impact on the adhesive mechanisms. According to [7], the highest adhesion can be enhanced using processes that result in a high micro-roughness ratio. On the other hand, a significant level of macro-roughness allows moderate adhesion. Surface with low micro- and macro-roughness provides the lowest durability of the connection.

### 3.2. Surface-Free Energy

**Figure 4:**
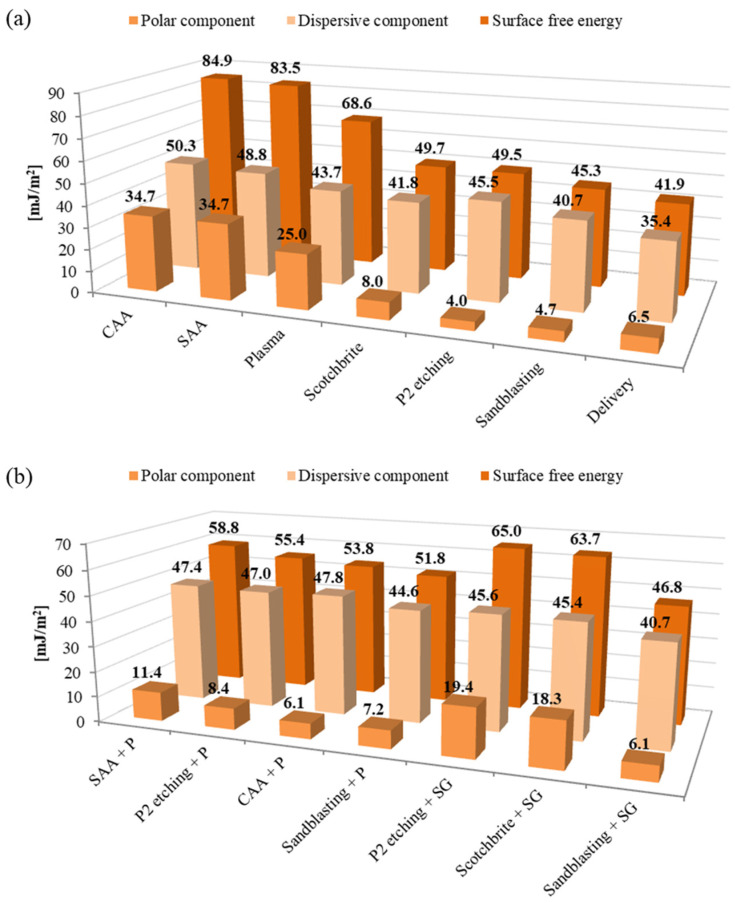
The values of surface-free energy and its components for aluminium surface.

**Figure 5:**
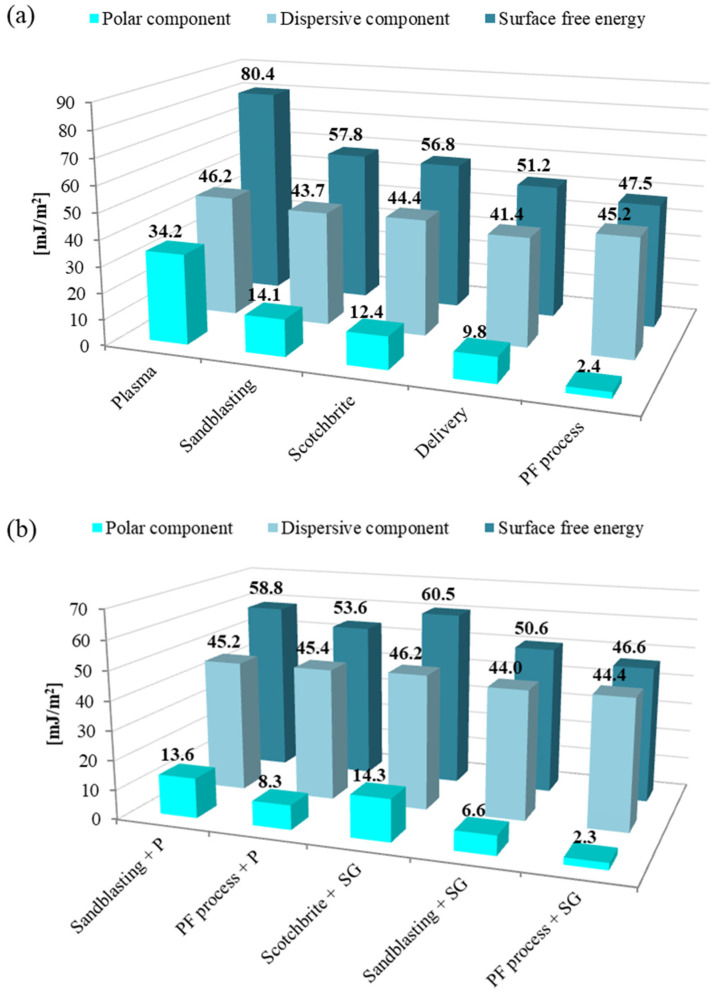
The values of surface-free energy and its components for titanium surface.

Figure 4 and Figure 5 shows the values of the surface free energy and its dispersion and polar components for various methods of aluminium and titanium surface preparation.

It was observed that the surface-free energy $\left(\gamma_{s}\right)$ depends on the method of the metal surface treatment. Moreover, the influence of the surface treatment method on the share of individual components—dispersive $\left(\gamma_{s}^{d}\right)$ and polar $\left(\gamma_{s}^{p}\right)$—was noted. The highest SFE was for aluminium, subjected to the electrochemical methods. For chromic acid anodizing $\gamma_{s}$ is 84.9 mJ/m^2^, and for sulphuric acid anodizing it is 83.5 mJ/m^2^. At the same time, for these methods, the highest values of the polar component (34.7 mJ/m^2^) concern other surface treatment methods. This is advantageous because it allows the formation of chemical bonds on the metal surface and increases the strength of the connection at the metal–composite interface. Lower, very similar values of $\gamma_{s}$ were for Scotch-Brite abrasion, P2 etching, and sandblasting and they are within the range (from 45.3 to 49.7 mJ/m^2^). For these treatments, the value of the polar component is significantly lower in comparison to the electrochemical treatment and amounts from 4.7 mJ/m^2^ to 8 mJ/m^2^. The intermediate surface free energy value was for plasma treatment (68.6 mJ/m^2^). For this method, a high value of the polar component was also noted (25.0 mJ/m^2^). Due to the application of this method, it is possible to obtain surfaces of high purity. It was confirmed by [34], where authors showed that plasma treatment allows for obtaining a surface free of impurities and at the same time activates chemical bonds on the metal surface.

For titanium, it was observed that the highest SFE value and polar component was after plasma treatment ($\gamma_{s}$ = 80.4 mJ/m^2^, $\gamma_{s}^{p}$ = 34.2 mJ/m^2^), in comparison to other surface treatment methods. The lowest surface free energy and polar component was noted for phosphate-fluoride process ($\gamma_{s}$ = 47.5 mJ/m^2^ and $\gamma_{s}^{p}$ = 2.3 mJ/m^2^); whereas, intermediate $\gamma_{s}$ values were observed for sandblasting, and Scotch-Brite abrasion, respectively, 57.8 mJ/m^2^ and 56.8 mJ/m^2^. Simultaneously, those surfaces had low polar components (from 12.4 mJ/m^2^ to 14.1 mJ/m^2^). 

As it was mentioned in the literature, the surface-free energy depends on various features, among others the geometric structure of the surface. The comparison of SFE results with average roughness and morphology shows that the relationship between those parameters is complex. It was noted that for aluminium, the highest surface roughness for sandblasting causes the lowest value of surface free energy. In a case where the grooves of a rough surface are filled with air, the water droplet cannot occupy the grooves, thereby leading to a two-phase system consisting of air and solid [45]. It is strongly connected with surface morphology. The highest SFE parameter was observed for CAA, which causes quite low roughness, however, this method guarantees a developed surface. Whereas in the case of titanium mechanical treatment methods, it causes high roughness parameter results in good surface energy parameters. Generally speaking, in most structural bonding applications the rate and extent of the interfacial contact between the adhesive and the adherent will increase with an increased surface roughness [23]. A rougher surface may help the thermodynamics of the wetting process and may also influence the increase in the adhesion strength. However, it is assumed that if the average roughness is less than 0.5 μm, it does not significantly affect wetting and surface free energy. 

Aluminium and titanium surfaces with the applied primer layer had a similar SFE value regardless of the applied surface treatment method (see Figure 4b and Figure 5b). The surface-free energy value for these surfaces ranges from 51.8 mJ/m^2^ to 58.5 mJ/m^2^ for both tested metal surfaces. The value of the polar component fluctuated between the limits from 7.2 mJ/m^2^ to 11.4 mJ/m^2^. This may indicate that a continuous, compact, and adequately dense primer layer was obtained on the tested surfaces, which mainly determines the physicochemical properties obtained.

In the case of the sol-gel coating, its influence on the physicochemical properties of the surface was noted. It was observed that the surface with sol-gel coating after P2 etching and after Scotch-Brite abrasion results in the $\gamma_{s}$ and $\gamma_{s}^{p}$ at the same level (see Figure 4b and Figure 5b). This indicates that due to the use of the sol-gel coating, it will be possible to obtain a surface capable of forming strong chemical bonds with the epoxy resin of the composite, which confirms [38,39,41]. This is different after sandblasting of aluminium and titanium, or also after phosphate-fluoride process, because the values of the surface-free energy, or the polar component, are at a much lower level. According to the authors of this study, it may be related to the surface topography and the possibility of penetration of unevenness and covering the unevenness with a thin sol-gel layer. Authors [46] showed that the decrease in the SFE is connected with the increase in the surface roughness due to ridges, asperities, and peaks restricting the spread of a droplet, thus the primer is not able to fully cover the surface. The above considerations will be developed in more detail in the next chapter about metal surface structure depending on the different surface treatment methods.

One of the crucial points for tailoring the interface adhesion strength is the wettability between metal and composite materials. The surface morphology evolution is considered to influence the surface roughness and further the water transport performance [47]. A solid surface becomes wet only when its free energy increases [45]. According to the theory of adhesion by wetting, the greater the surface energy of the substrate (in comparison to that of the adhesive), the greater the wettability of the surface, and consequently, the greater the degree of adhesive-substrate adhesion [10]. Generally, three basic models govern wetting under different scenarios—Wenzel’s model, Young’s model, and Cassie–Baxter’s model.

Xu et al. [27] observed that due to different mechanisms of resin infiltration, increasing roughness and surface free energy are not always suitable approaches to achieving high adhesive bonding. Authors [48] noted that mechanical treatment does not create very good conditions for mechanical interlocking, nor does it lead to a surface morphology with appropriate unevenness (cavities). It is mainly of a cleaning nature in order to remove the weak boundary layer, most often impurities, from the surface, contributing to the maintenance of appropriate thermodynamics and wetting kinetics [48]. Proper topography and surface morphology for mechanical bonding can be obtained by applying chemical surface treatment methods, e.g., etching [48]. Authors [27] noted that epoxy can easily infiltrate into the pits, however, it cannot always infiltrate into the nanometre scale pores because the size of the pores can affect the wetting behaviour. The capillary mechanism of the pores may explain the process of infiltrating the metal surface with resin. Capillary wetting of the pore surface is dependent on the ratio of the pore diameter to the pore spacing and the ratio of the pore depth to the pore diameter. Therefore, the capillary wetting mechanism changes with the microstructure. Epoxy can not only penetrate the cavities but also penetrate the pores on a nanometric scale under the action of capillary force.

### 3.3. Surface Morphology

**Figure 6:**
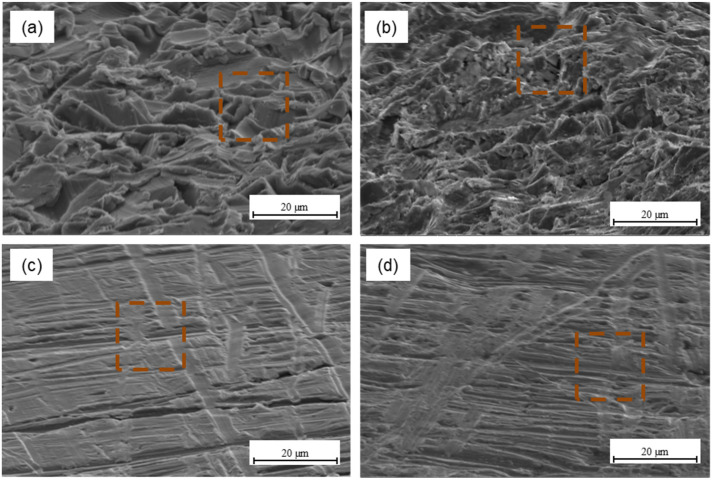
The surface of aluminium after sandblasting (**a**) and Scotch-Brite abrasion (**c**), and titanium after sandblasting (**b**) and Scotch-Brite abrasion (**d**).

The characteristic morphology of aluminium and titanium surfaces subjected to the mechanical methods, such as sandblasting and Scotch-Brite abrasion, is shown in the Figure 6.

It was observed that aluminium and titanium surface after sandblasting is characterized by rises and grooves, which are randomly oriented. This results in obtaining a high surface roughness, which was confirmed by the stereometric rates (see Table 2 and Table 3). For Scotch-Brite abrasion of aluminium and titanium surface (see Figure 6c,d) some characteristic scratches oriented in the direction of the Scotch-Brite pad use movement were observed. The structure is characterized by low roughness and low-surface irregularities, compared to other surface treatments (see Table 2 and Table 3). It was noted that the surface is relatively smooth and the treatment mainly serves to remove the existing oxide layer, and thus cleans the surface.

**Figure 7:**
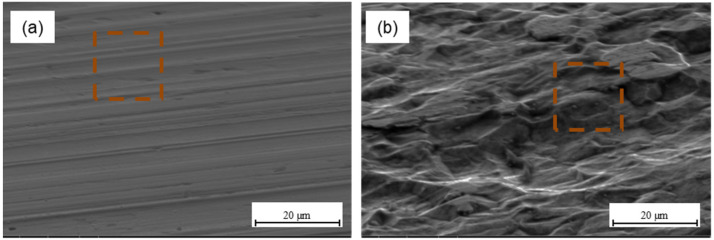
The surface of aluminium (**a**) and titanium (**b**) after plasma treatment.

Mechanical methods are commonly used in the metal surface treatment for bonding. According to the theory of mechanical adhesion, surface irregularities allow mechanical interlocking for increasing adhesion at the metal–composite interface. Mechanical interlocking occurs when the adhesive can penetrate the surface irregularities, and it becomes mechanically blocked with the substrate [49]. Literature data show that a rougher surface is desirable due to the fact that provides a stronger internal adhesion, which is associated with an increase in surface energy and guarantees an effective contact at the metal–composite interface, which in turn activates the mechanical keying mechanism [13,50]. At the same time, a necessary condition is the production of a certain shape of irregularities that will be capable to hold the adhesive [23]. It is possible due to the appropriate surface roughness as a result of the mechanical methods. An increase in the total surface enables more reactions between the metal and the adhesive. However, based on the other authors’ results, it seems that in the case of sandblasting, when a certain roughness is exceeded, shear strength decreases because of roughness being too high, which blocks the effective ingress of the resin, causing it to settle on the peaks [51,52,53]. Optimum surface roughness allows obtaining high adhesion [52]. Figure 7 presents the morphology of aluminium (Figure 7a) and titanium surface (Figure 7b) after plasma treatment.

**Figure 8:**
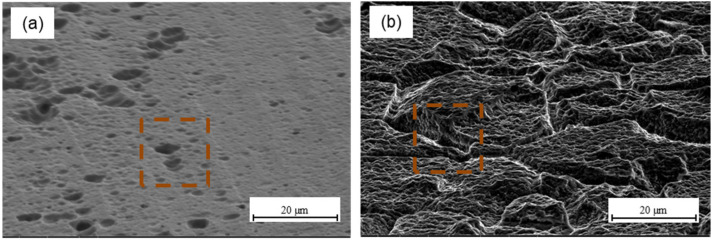
Aluminium surface morphology after P2 etching (**a**) and titanium after phosphate-fluoride process (**b**).

The morphology analysis after plasma treatment indicates that the surface is smooth. It was observed that the use of atmospheric-pressure plasma, in comparison to the delivery state, changes neither the surface topography nor the surface roughness (see Table 2 and Table 3). However, it cleans the metal surface from contamination and oxides the surface, with the generation of polar hydroxyl groups. Those groups promote strong chemical bonding due to covalent bonding between hydroxyl groups and the epoxy and may influence the increase in adhesion strength [35]. This is reflected in the SFE tests, where very high-surface free-energy values were achieved for both aluminium and titanium, particularly the polar component (see Figure 4a and Figure 5a). The characteristic morphology after chemical methods were shown in Figure 8.

The morphology of aluminium after alloy P2 etching (see Figure 8a) indicates that the surface is homogenous with high porosity. Moreover, it has low roughness and numerous micro-irregularities, which was confirmed by the determined stereometric parameters (see Table 2) and AFM analysis (see Figure 3b). Moreover, the presence of randomly oriented digestive pits and cavities was observed. They can be places for resin penetration and influence the achievement of adequate adhesion, which is confirmed by work [23]. The author noted that chemical treatment may create surface irregularities that enable mechanical keying. Prolongo et al. [21] noted that the surface after etching is more rugged and bumpy, in comparison to Scotch-Brite abrasion. The P2 etching process may be an alternative to chromic acid anodizing; it produces surfaces that are receptive to adhesives. This treatment can improve adhesion through the chemistry surface modification and a generation of the strong adhered porous oxide layer, which provides a better mechanical interlocking with the epoxy [38]. The surface morphology after phosphate-fluoride process reveals clear grain boundaries, which were formed as a result of strong etching of the surface (see Figure 8b). In the areas of the grain, characteristic surface irregularities in the form of numerous depressions and crevices are visible. According to [7,20,44], as a result of phosphate-fluoride process, a continuous and thin oxide layer arises, which has an anatase structure, and its thickness is estimated at about 8 nm.

**Figure 9:**
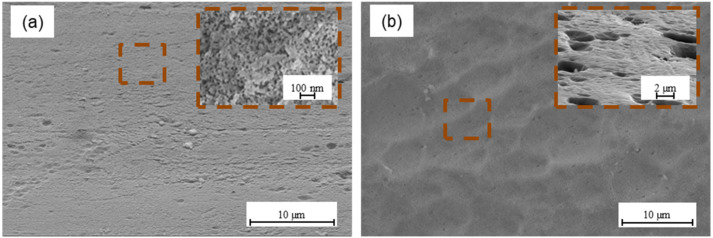
Aluminium surface morphology after chromic acid anodizing (**a**), and sulphuric acid anodizing (**b**).

The characteristic morphology of the aluminium surface subjected to electrochemical methods, such as chromic acid anodizing and sulphuric acid anodizing, is shown in Figure 9a,b, respectively.

**Figure 10:**
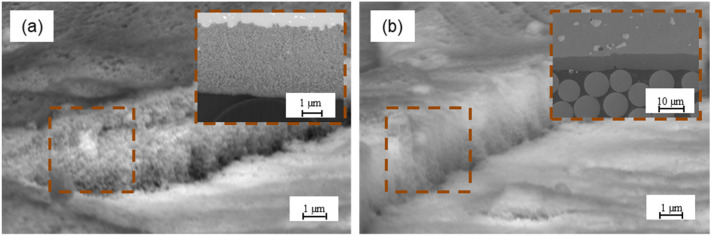
Fracture of oxide layer after chromic acid anodizing (**a**) and sulphuric acid anodizing (**b**).

The obtained oxide layers after the anodizing processes are characterized by a homogeneous structure with characteristic pores that are evenly spaced. On the oxide layer obtained after chromic acid anodizing, some characteristic whiskers were observed, which most likely resulted from the previous pre-treatment during the etching stage. The surface shows a large number of micro-irregularities, as well as a low level of roughness (see Table 2 and Figure 3f). Such a surface increases the contact area, enables the formation of the interfacial area, as well as the redistribution of stresses on the metal–composite interface, thanks to which it is possible to create both a mechanical and chemical connection. A suitable transition layer obtained by anodizing is extremely important for improving adhesion at the metal–composite interface in FMLs [54,55]. In the case of oxide layers after sulphuric acid anodizing, the surface has a non-homogeneous porous structure with the presence of areas with the significant geometrical diversity of the layer, and it is close to the scaly one (see Figure 9b). It was observed that the SAA process causes higher roughness and a low amount of micro-roughness, in comparison to chromic acid anodizing (see Table 3). Figure 10 shows the fracture of aluminium oxide layer after electrochemical methods.

The observations indicate significant differences in the porous structure of the oxide layers (see Figure 10). It was noted that the oxide layers after chromic acid anodizing are characterized by significantly larger pore sizes, in comparison to the pores obtained by sulphuric acid anodizing. The pore size ranges from 20 nm to 32 nm (an average 25 nm ± 5 nm) for oxide layers after chromic acid anodizing and from 8 nm to 12 nm (an average 9 nm ± 1 nm) for layers produced in sulphuric acid anodizing. The number of pores for the oxide layer in chromic acid anodizing is on average 201 × 10^6^/mm^2^, while the number of pores for the oxide layer in sulphuric acid anodizing is on average 662 × 106/mm^2^. Literature data show that the smaller the pore size, although more difficult pore filling to adhesive have greater adhesive joint strength. When the pore size is larger, the adhesive joint strength is lower, despite more easily filling the pores [56].

**Figure 11:**
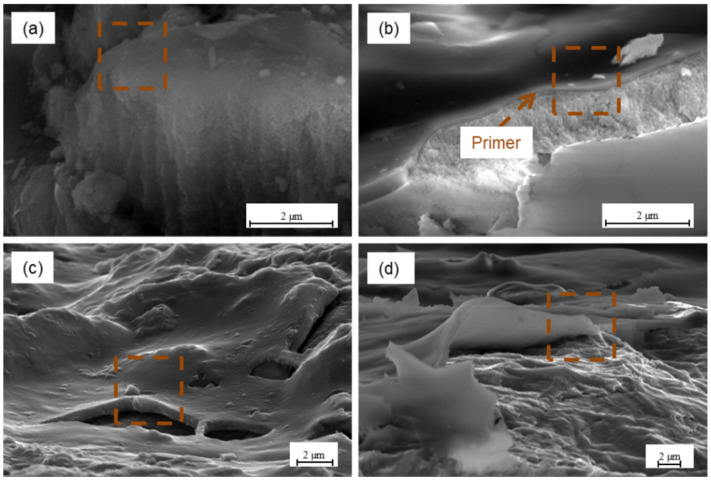
Aluminium surface with primer after chromic acid anodizing (**a**), sulphuric acid anodizing (**b**), and titanium after sandblasting (**c**), and phosphate-fluoride process (**d**).

The analysis of the oxide layers (see Figure 10) shows that anodizing allows for obtaining a relatively thin and smooth layer. The thickness of the oxide layers is uniform over the entire surface, after the CAA process is equal to 4 ± 0.3 μm, while after SAA process it is 6 ± 0.2 μm. The oxide layers obtained in the chromic and sulphuric acid anodizing are characterized by an irregular structure with a column orientation with pores located at different angles to the surface. The structure of the oxide layers can be characterized as spongy. This structure has numerous irregularities and porosities but simultaneously exhibits a high degree of uniformity. Such a porous surface increases the contact area. Moreover, it allows the penetration of various substances, making the surface more wettable and characterized by high surface energy. In these tests, the highest surface-free energy and polar components were obtained for the surface after chromic acid anodizing (see Figure 4a). Figure 11 shows the fracture layer of aluminium and titanium after different surface treatments with a primer coating.

**Figure 12:**
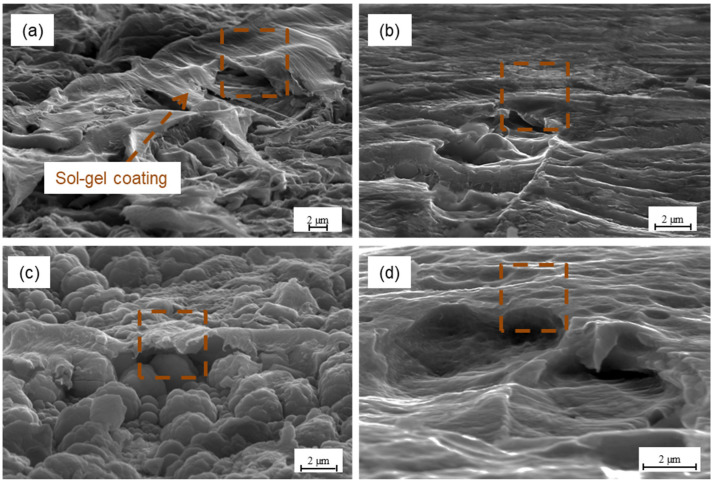
The titanium surface with sol-gel layer after sandblasting (**a**), phosphate-fluoride process (**c**), and aluminium after Scotch-Brite abrasion (**b**), P2 etching (**d**).

It was observed that the structure obtained after different surface methods has a significant effect on the transition layers of the primer or sol-gel type. It was noted that the pores after CAA treatment are of appropriate size and that the primer filled them. This is contrary to sulphuric acid anodizing, where the pores size does not allow for the filling. In the case of chromic acid anodizing (see Figure 11a), there was no separate primer layer on the surface and no visible separation boundary between the oxide layer and the primer. Whereas for SAA treatment and other methods, the primer layer is visible. The analysis revealed that the primer layer is separate and thin, approximately 0.47 µm (±0.23) (see Figure 11b–d). It was noted that the primer covered surface irregularities (see Figure 11c). After the phosphate-fluoride process, the primer coated surface irregularities and filled the boundary channels of the titanium grains, creating a thin layer on the surface (see Figure 11d). Due to the appropriate (low) viscosity and achieving good wettability of the primer-oxide layer system. The primer can spread over the surface, obtaining the largest possible real contact area. Moreover, it can displace air and other contaminants (which may help to prevent the formation of porosity), as well as penetrate and fill the porosity oxide layer. As it can be seen, the primer layer will have a decisive influence on the physicochemical properties (SFE), where an almost constant value was noted. Figure 12 shows the surfaces of aluminium and titanium with a sol-gel coating applied.

The sol-gel layer, due to the characteristics of the preparation, creates a smooth and very thin layer on the surface of about 0.10 µm (±0.01), (see Figure 12). It fills in the irregularities of the substrate well, also gaps and porosity, thanks to which its use increases the SFE (see Figure 4b and Figure 5b). However, with higher surface roughness, the sol-gel is not able to completely cover its irregularities. In such cases, no increase in the SFE and its components was observed. It was noted after sandblasting process and the phosphate fluoride process, compared to other surface treatments. In addition, it is probably associated with the above-mentioned high surface roughness. Then, a thin layer of sol-gel only penetrates the low rises and flows into the pits, while the peaks of high roughness remain uncovered (see Figure 4b and Figure 5b). Such a phenomenon was not noted in the case of the primer because the layer has a greater thickness and it is able to cover larger surface irregularities. It was observed that for aluminium after P2 etching, the sol-gel coating filled the micro-irregularities, and, as a result, good surface-free energy (see Figure 4a) and a high polar component were observed. In this case, the surface is advantageously filled with the possibility of being influenced not only by chemical bonds but also by a mechanical connection. The use of sol-gel layers results in the creation of organic-inorganic coating and the formation of strong chemical–covalent bonding and the silane function groups creation, for example, epoxy or resin adhesives and amine groups [33,42].

**Figure 13:**
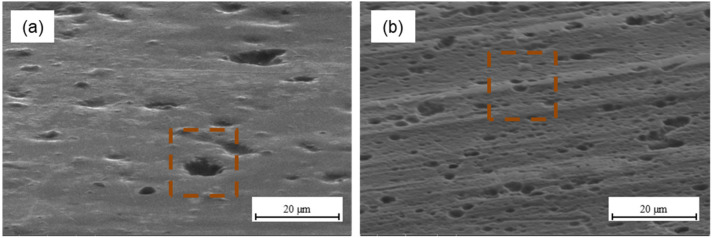
Aluminium after P2 etching with primer (**a**) and sol-gel layer (**b**).

Figure 13 shows the comparison of the primer layer and sol-gel coating on aluminium surface after P2 etching.

The use of a sol-gel coating may have a positive effect on the specificity of the surface. By filling its unevenness and porosity with a thin layer, from the point of view of adhesion at the boundary of the separation. Providing the possibility of obtaining a hybrid mechanical-chemical connection. The sol-gel has been noted to soak into the surface leaving a thin layer as opposed to the primer layer (see Figure 13). 

## 4. Conclusions

The paper presents the issues of aluminium and titanium surface treatments using mechanical, chemical, and electrochemical methods in terms of the possibility of obtaining high adhesion at the metal–composite interface. Relationships that may directly affect the properties of adhesive joints, and thus the possibility of shaping the metal–composite interface in FMLs have been shown.

1. The applied methods of surface treatment results in a structure with specific geometric, morphological, and physicochemical characteristics.
2. Mechanical methods (sandblasting or Scotch-Brite abrasion) create a characteristic geometric structure with a high roughness, especially after sandblasting; it ranges from 0.78 to 1.18 µm and changes the surface topography. These methods increase the surface area but do not obtain the appropriate shape of the inequalities guaranteeing mechanical interlocking and adhesive anchoring. However, with good wettability at the same time, it can lead to good tangible adhesion.
3. Plasma techniques accurately clean the surface, but do not change its topography. It has low roughness approx. 0.19 µm (aluminium), and 0.38 µm (titanium). Therefore, these methods will have limited application to maintain high adhesion at the metal–composite interface.
4. In terms of the expected suitable adhesion properties, the most advantageous is still the aluminium treatment by creating oxide layers in the anodizing process, especially chromic acid anodizing. Then, the surface has a homogeneous structure with a high level of micro irregularities and appropriate physicochemical properties (SFE). It ranges from 84.9 mJ/m^2^ for CAA and 83.5 mJ/m^2^ for SAA.
5. The beneficial structure was obtained after chemical etching. Appropriate topography and morphology was created. Particularly for aluminium after P2 etching, low roughness *R_a_* = 0.07 µm was noted, with numerous micro-irregularities and the presence of porosity. This surface modification may be a combination of mechanical and chemical influence. In these cases, the key influence may be the specific interaction between the surface and the transition layers.
6. The above modifications of the surfaces cause a synergistic interaction with the intermediate layers (primer and sol-gel). The structure of the top layer allows penetration by the primer, and particularly by the sol-gel. The porous structure with the increased interfacial surface area may favour the penetration by the primer or particularly the promising sol-gel. As a result, such modification can influence chemical and mechanical mechanisms.

## Figures and Tables

**Table 1:** Methods of surface treatments.

Surface Treatment	Acronym	Parameters Description
Scotch-Brite abrasion	SC	The process was conducted manually using an abrasive Scotch-Brite disc pad 07447+ (3M, Saint Paul, MN, USA). The process was relayed on the moving Scotch-Brite pad from side-to-side and then changed the direction by 90 degrees until a cross coat was obtained. Then, the surface was precisely degreased with acetone using lint-free tissue.
Sandblasting	S/B	The sandblasting process was conducted with alumina (Al_2_O_3_) powder. The powder was 180 µm grits. Then, the surface was precisely degreased with acetone using lint-free tissue.
P2 etching	E	The process was carried out according to Russell and Garnis, the etchant contained concentrated sulphuric acid, ferric sulphate, and sufficient water. The aluminium surface was etched for 11 min at a temperature from 63 °C to 65 °C, then rinsed in a water tank and after that drained.
Phosphate-Fluoride process	PF	The process included degreasing, rinsing, as well as digestion with hydro-fluoric acid, nitric acid and sodium sulphate for 2–3 min at room temperature 24 °C. In addition, phosphate-fluoride treatment with sodium phosphate, potassium fluoride, and hydrofluoric acid was carried out for 1.5–2.5 min. The process temperature was 24 °C.
Chromic acid anodizing	CAA	The process included the following stages: alkaline degreasing, rinsing, and etching in a sulphochrome bath. Chromic acid anodizing was prepared using chromic acid anhydride CrO_3_. The process temperature was 38–42 °C, voltage: 20 V, time: 45 min.
Sulphuric acid anodizing	SAA	The process included etching with sodium hydroxide NaOH, rinsing, brightening with nitric acid HNO_3_, filling with potassium dichromate K_2_Cr_3_O_7_, or drying. The sulphuric acid anodizing was performed in sulphuric acid H_2_SO_4_, process temperature was 10–15 °C, voltage: 13–24 V, time: 24 min.
Plasma	K	The plasma process was conducted under the following conditions: power: 140 W, He flow: 30 L/min, O2 striking flow: 0.1 L/min. The plasma activation was per-formed using the Atomflo 500 plasma systems (Surfx Technologies).
Sol-gel coating	SG	The sol-gel coatings were produced using the 3M™ Surface Pre-Treatment AC 130-2 (3M™) two-component formulation. Two components were mixed and left on induction time of 30 min. Then, the mixture was applied to the surface using lint-free tissue, it was left to drain for 10 min and after that, the coated surface was left for drying at room temperature for 60 min.
Primer	P	The aluminium surface was coated with EC-3924B corrosion-inhibiting structural adhesive primer (3M™ Scotch-Weld™). The titanium surface was coated with EC-3960 (3M™ Scotch-Weld™). The process consists of applying a thin layer of a primer using the spray method.

**Table 2:** Roughness parameters for aluminium depending on the different surface treatments.

Surface Treatment	Roughness Parameters [µm]					
	*R_a_*	*R_q_*	*R_t_*	*R_z_*	*R_v_*	*R_p_*
Sandblasting	0.78 (±0.09)	0.98 (±0.10)	4.97 (±0.16)	4.96 (±0.16)	2.26 (±0.44)	2.70 (±0.28)
Sulphuric acid anodizing	0.39 (±0.03)	0.51 (±0.06)	3.01 (±0.69)	2.83 (±0.44)	1.89 (±0.37)	0.95 (±0.06)
Aluminium degreased	0.21 (±0.00)	0.24 (±0.01)	1.11 (±0.21)	1.08 (±0.19)	0.63 (±0.12)	0.46 (±0.07)
Chromic acid anodizing	0.19 (±0.01)	0.24 (±0.01)	1.43 (±0.06)	1.43 (±0.06)	0.86 (±0.11)	0.57 (±0.17)
Scotch-Brite abrasion	0.19 (±0.00)	0.23 (±0.01)	1.22 (±0.12)	1.20 (±0.09)	0.67 (±0.09)	0.52 (±0.00)
Plasma	0.17 (±0.01)	0.31 (±0.00)	2.84 (±0.23)	2.69 (±0.03)	1.48 (±1.13)	1.21 (±1.17)
P2 etching	0.07 (±0.04)	0.13 (±0.10)	1.45 (±0.71)	1.32 (±0.89)	1.63 (±0.11)	0.19 (±0.06)

**Table 3:** Roughness parameters for titanium depending on the different surface treatments.

Surface Treatment	Roughness Parameters [µm]					
	*R_a_*	*R_q_*	*R_t_*	*R_z_*	*R_v_*	*R_p_*
Sandblasting	1.14 (±0.18)	1.45 (±0.18)	7.54 (±0.55)	3.31 (±1.06)	4.23 (±0.52)	7.54 (±0.55)
Phosphate–fluoride process	0.98 (±0.08)	1.24 (±0.08)	6.44 (±0.51)	3.27 (±0.66)	3.16 (±0.58)	6.43 (±0.52)
Plasma	0.38 (±0.04)	0.54 (±0.06)	3.53 (±0.28)	0.95 (±0.01)	2.58 (±0.29)	3.53 (±0.28)
Titanium degreased	0.32 (±0.01)	0.43 (±0.04)	2.99 (±0.16)	1.02 (±0.11)	1.71 (±0.53)	2.73 (±0.42)
Scotch-Brite abrasion	0.28 (±0.03)	0.38 (±0.07)	2.24 (±0.69)	0.60 (±0.02)	1.64 (±0.66)	2.24 (±0.69)

## Data Availability

The data presented in this study are available on request from the corresponding author.
